# The Positive Lymph Node Ratio Predicts Survival in T_1−4_N_1−3_M_0_ Non-Small Cell Lung Cancer: A Nomogram Using the SEER Database

**DOI:** 10.3389/fonc.2020.01356

**Published:** 2020-08-05

**Authors:** Yi Liao, Guofang Yin, Xianming Fan

**Affiliations:** ^1^Department of Respiratory and Critical Care Medicine, The Affiliated Hospital of Southwest Medical University, Luzhou, China; ^2^Inflammation & Allergic Diseases Research Unit, The Affiliated Hospital of Southwest Medical University, Luzhou, China

**Keywords:** nomogram, positive lymph node, non-small cell lung cancer, SEER, prognosis

## Abstract

**Background:** An increasing number of studies have shown that the positive lymph node ratio (pLNR) can be used to evaluate the prognosis of non-small cell lung cancer (NSCLC) patients. To determine the predictive value of the pLNR, we collected data from the Surveillance, Epidemiology, and End Results (SEER) database and performed a retrospective analysis.

**Methods:** We collected survival and clinical information on patients with **T_1−4_N_1−3_M_0_** NSCLC diagnosed between 2010 and 2016 from the SEER database and screened them according to inclusion and exclusion criteria. X-tile software was used to obtain the best cut-off value for the pLNR. Then, we randomly divided patients into a training set and a validation set at a ratio of 7:3. Pearson's correlation coefficient, tolerance and the variance inflation factor (VIF) were used to detect collinearity between variables. Univariate and multivariate Cox regression analyses were used to identify significant prognostic factors, and nomograms was constructed to visualize the results. The concordance index (C-index), calibration curves, and decision curve analysis (DCA) were used to assess the predictive ability of the nomogram. We divided the patient scores into four groups according to the interquartile interval and constructed a survival curve using Kaplan–Meier analysis.

**Results:** A total of 6,245 patients were initially enrolled. The best cut-off value for the pLNR was determined to be 0.55. The nomogram contained 13 prognostic factors, including the pLNR. The pLNR was identified as an independent prognostic factor for both overall survival (OS) and cancer-specific survival (CSS). The C-index was 0.703 (95% CI, 0.695–0.711) in the training set and 0.711 (95% CI, 0.699–0.723) in the validation set. The calibration curves and DCA also indicated the good predictability of the nomogram. Risk stratification revealed a statistically significant difference among the four groups of patients divided according to quartiles of risk score.

**Conclusion:** The nomogram containing the pLNR can accurately predict survival in patients with **T_1−4_N_1−3_M_0_** NSCLC.

## Introduction

As the global population ages, cancer is becoming an increasing burden on human health. The World Health Organization's International Agency for Research on Cancer (IARC) released its broad survey of cancer morbidity and mortality worldwide in 2018 and showed that lung cancer led the list of new cases and deaths worldwide in that year. Among all the pathological types of lung cancer, non-small cell lung cancer (NSCLC) is the most common type, accounting for 80% of all lung cancers (1, 2). In recent years, the death rate of lung cancer patients in the United States has decreased gradually, whereas that in China is still increasing. The incidence of lung cancer among non-smokers in China is significantly higher than that in the United States, especially among women (3). The global cancer situation remains very serious.

Lung cancer, as a heterogeneous disease, should be treated as an individual entity. According to this idea, the latest (8th edition) staging system for tumor lymph node metastasis (TNM) can more accurately predict the prognosis of NSCLC patients than the 7th edition (4). However, the 8th edition of the N staging system, which divides lymph nodes into four groups based on their anatomical area, has changed little since the 7th edition (5). However, staging based solely on the anatomical region of lymph nodes cannot avoid the problem of lymph node heterogeneity and is insufficient for clinical application.

The latest TNM staging system considers the grouping of anatomical regions and the number of positive lymph nodes for partial tumor staging [e.g., gastric cancer (6) and rectal cancer (7)]. With continuous improvements in detection methods, the number of lymph nodes and the positive lymph node ratio (pLNR) as the bases for lymph node staging have attracted increasing attention. In addition, the pLNR has been shown to predict the prognosis of patients with pancreatic cancer (8), breast cancer (9), and laryngeal squamous cell carcinoma (10).

The Surveillance, Epidemiology, and End Results (SEER) database (https://seer.cancer.gov/) is the authoritative cancer statistics database in the United States. The SEER database has a large sample size and samples from multiple populations, which makes studies based on the SEER database of high clinical value. In this study, to investigate whether the pLNR can predict the prognosis of non-advanced NSCLC patients, we collected patient and clinical information from the SEER database and conducted a large-sample retrospective study.

## Materials and Methods

### Data Collection

Data were extracted from the SEER database with SEER^*^Stat Software (version 8.3.6; https://seer.cancer.gov/data-software/), and the Incidence SEER 18 Regs Custom Data (with additional treatment fields) and Nov 2018 Sub (1973-2016 varying) datasets were selected for analysis (username for log in: 14112-Nov2018). The NSCLC patients in our limited group were diagnosed with **T_1−4_N_1−3_M_0_** between 2010 and 2016. The corresponding selection formula in the software was expressed as follows: {Site and Morphology. TNM 7/CS v0204+ Schema}=“Lung” AND {Stage-American Joint Committee on Cancer (AJCC). Derived AJCC Stage Group, 7th ed. (2010+)}=“I,” “IA,” “IB,” “II,” “IIA,” “IIB,” “III,” “IIIA,” “IIIB” AND {Race, Sex, Year Dx, Registry, County, Year of diagnosis}= “2010,” “2011,” “2012,” “2013,” “2014,” “2015,” “2016.” The extracted clinical information included the following: patient ID, age at diagnosis, sex, grade, laterality, primary site, histologic type, T stage, N stage, surgery at the primary site, scope of regional lymph node surgery radiation recode, radiation sequence with surgery, chemotherapy recode, regional nodes examined, regional nodes positive, survival months, vital status recode, SEER cause-specific death classification, marital status at diagnosis, insurance recode, first malignant primary indicator, sequence number and diagnostic confirmation.

### Data Processing

Samples meeting any of the following criteria were excluded: (1) missing or unknown clinical patient information; (2) pathological tumor of type small cell carcinoma, sarcoma or another type not belonging to NSCLC; (3) patient survival time less than or equal to 0 months; (4) fewer than 1 regional nodes examined or operation not involving lymph node removal; (5) patient receipt of preoperative or intraoperative radiotherapy; (6) multiple primary cancers; (7) a first malignant primary indicator entry of “yes” and a sequence number of one (primary only); (8) diagnostic confirmation not obtained via positive histology and diagnosis obtained through a death certificate or autopsy; and (9). AJCC stage not corresponding to **T_1−4_N_1−3_M_0._**

After filtering the data, additional classification was performed. Age was treated as a continuous variable, and the other factors were treated as categorical variables. Patients who were widowed, divorced, unmarried or single or had a domestic partner (unmarried) were all considered unmarried. We also divided the pathological tissue types into adenocarcinoma (ADC), squamous cell carcinoma (SCC), adenosquamous carcinoma (ASC) and large cell carcinoma (LCC). Other histopathologic types, such as giant cell carcinoma and spindle cell carcinoma, were classified as “other.” Overall survival (OS) was defined as the time from the beginning of random assignment to death caused by any reason. Cancer-specific survival (CSS) was defined as the time from the beginning of random assignment to death caused by cancer.

The formula used to calculate the pLNR was regional nodes positive/regional nodes examined. In most clinical studies, the correlations between continuous variables and outcomes are not linear, and continuous variables are not as convenient as categorical variables in clinical applications. For general dichotomized outcome indicators, Youden's index can be calculated, but for survival-type data, it is difficult to obtain truncated values. X-tile software was used to determine the optimal cut-off value of the survival data (11). This software analyzes different values as cut-off values for a statistical test, and the result with the smallest *p*-value is considered the best cut-off value. Patients were divided into high-pLNR and low-pLNR groups according to the optimal cut-off value.

### Construction and Validation of the Nomogram

We randomly divided the enrolled patients into a training set and a validation set at a ratio of 7:3, and the clinical prognosis information of the two groups of patients was analyzed. For the training set, a univariate Cox regression analysis was used, and after excluding the prognostic factors with no statistical significance, the remaining factors were included in a multivariate Cox regression analysis. The hazard ratio and 95% confidence interval (CI) were also calculated. Finally, according to the same exclusion criteria, we obtained the final factors that affected the prognosis of non-advanced NSCLC patients. Based on the multivariate Cox regression analysis, the multiprediction indexes were integrated to further express the relationships between the variables in the prediction model. The rms (12), foreign and survival packages in R software were used to construct the nomogram.

To verify the prediction accuracy of the nomogram, we calculated Harrell's concordance index (C-index) (13) and calibration curves for the training and validation groups. In addition, decision curve analysis (DCA) performed with the DecisionCurve package (14) was used to determine the 1-, 3-, and 5-year survival rates of the two sets. DCA was performed to compare AJCC TNM stage (7th edition), the pLNR and the nomogram.

### Risk Stratification

To clarify the descriptive power of the nomogram, we obtained the risk scores based on the clinical factors in the nomogram and calculated the score of each patient in the validation set. Then, we divided the validation set into four groups according to quartile spacing, constructed the survival curve using Kaplan–Meier analysis and calculated the *p*-value with the log-rank test. The survival curves of NSCLC patients for OS and CSS in the training set and validation set were constructed in the same manner.

### Statistical Analysis

All the statistical tests were performed using R (version 3.6.0, https://www.r-project.org) and SPSS software (version 22.0; IBM Corp., Armonk, NY, USA). Cumulative survival time (for both OS and CSS) was calculated using the Kaplan-Meier method, and the differences in survival curves were analyzed using the log-rank test. We calculated Pearson's correlation coefficients to detect collinearity among the variables. A correlation coefficient of <0.7 between two independent variables was considered indicative of no multicollinearity (15). We also calculated tolerance and variance inflation factor (VIF) values to evaluate multicollinearity between variables, with tolerance <0.1 and VIF >10 considered indicative of multicollinearity. *P* < 0.05 was considered statistically significant (16).

## Results

### Patient Clinicopathological Characteristics

**Figure 1:**
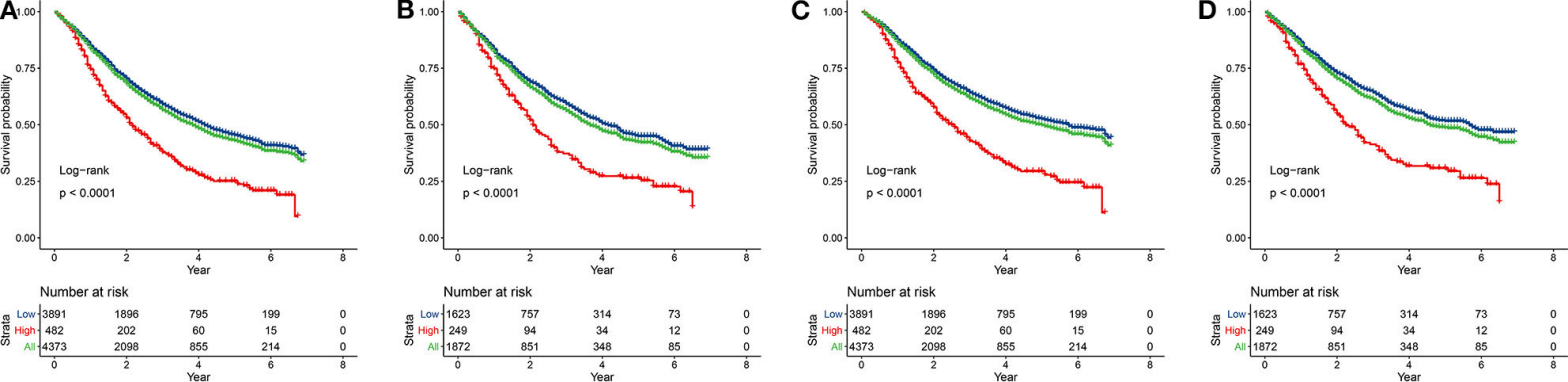
Kaplan-Meier curves of OS and CSS for patients with high and low pLNR in the training set [**(A)**, OS; **(C)**, CSS] and the validation set [**(B)**, OS; **(D)**, CSS]. OS, overall survival; CSS, cancer-specific survival; pLNR, positive lymph node ratio.

According to our inclusion and exclusion criteria, 6,245 patients with **T_1−4_N_1−3_M_0_** NSCLC were enrolled. The best cut-off value for the pLNR was determined to be 0.55. The clinicopathological characteristics of the patients in the training set and the validation set are shown in Table 1. Among the patients, 4,373 were allocated to the training set, and 1,872 were allocated to the validation set. The prognosis of patients with high pLNR was significantly worse than that of both patients with low pLNR and the overall patient population for both OS (Figures 1A,B) and CCS (Figures 1C,D).

**Table 1:** The demographics and pathological characteristics of the included patients in the training and validation sets.

**Variable (%)**	**SEER cohort**	
	**Training set**	**Validation set**
Age (year)	65.51 ± 10.42	65.66 ± 10.19
Sex		
Female	2,096 (47.9)	943 (50.4)
Male	2,277 (52.1)	929 (49.6)
Race		
White	3,567 (81.6)	1,527 (81.6)
Black	512 (9.4)	184 (9.8)
Other	394 (9.0)	161 (8.6)
Marital status		
Unmarried	1,734 (39.7)	766 (40.9)
Married	2,639 (60.3)	1,106 (59.1)
Insurance status		
Uninsured	105 (2.4)	56 (3.0)
Insured	4,268 (97.6)	1,816 (97.0)
Laterality		
Left	1,940 (44.4)	879 (47.0)
Right	2,433 (55.6)	993 (53.0)
Primary site		
Main bronchus	70 (1.6)	37 (2.0)
Upper lobe	2,383 (54.5)	1,013 (54.1)
Middle lobe	234 (5.4)	102 (5.4)
Lower lobe	1,579 (36.1)	674 (36.0)
Overlapping lesion	107 (2.4)	46 (2.5)
Grade		
I	337 (7.7)	135 (7.2)
II	1,934 (44.2)	853 (45.6)
III	2,035 (46.5)	840 (44.9)
IV	67 (1.5)	44 (2.4)
Histology		
ADC	2,538 (58.0)	1,079 (57.6)
SCC	1,209 (27.6)	502 (26.8)
ASC	144 (3.3)	82 (4.4)
LCC	83 (1.9)	35 (1.9)
Other	399 (9.1)	174 (9.3)
T stage		
T1	1,062 (24.3)	460 (24.6)
T2	2,234 (51.1)	962 (51.4)
T3	830 (19.0)	351 (18.8)
T4	247 (5.6)	99 (5.3)
N stage		
N1	2,557 (58.5)	1,081 (57.7)
N2	1,784 (40.8)	772 (41.2)
N3	32 (0.7)	19 (1.0)
Surgery		
Lobectomy	3,822 (87.4)	1,638 (87.5)
Pneumonectomy	551 (12.6)	234 (12.5)
Lymph node dissection		
1 to 3	254 (5.8)	132 (7.1)
4 and more	4,119 (94.2)	1,740 (92.9)
Chemotherapy		
No	1,452 (33.2)	324 (33.3)
Yes	2,921 (66.8)	1,248 (66.7)
Radiotherapy		
No	3,366 (77.0)	1,460 (78.0)
Yes	1,007 (23.0)	412 (22.0)
pLNR		
Low	3,891 (89.0)	249 (86.7)
High	482 (11.0)	1,623 (13.3)

### Cox Regression Analysis

**Figure 2:**
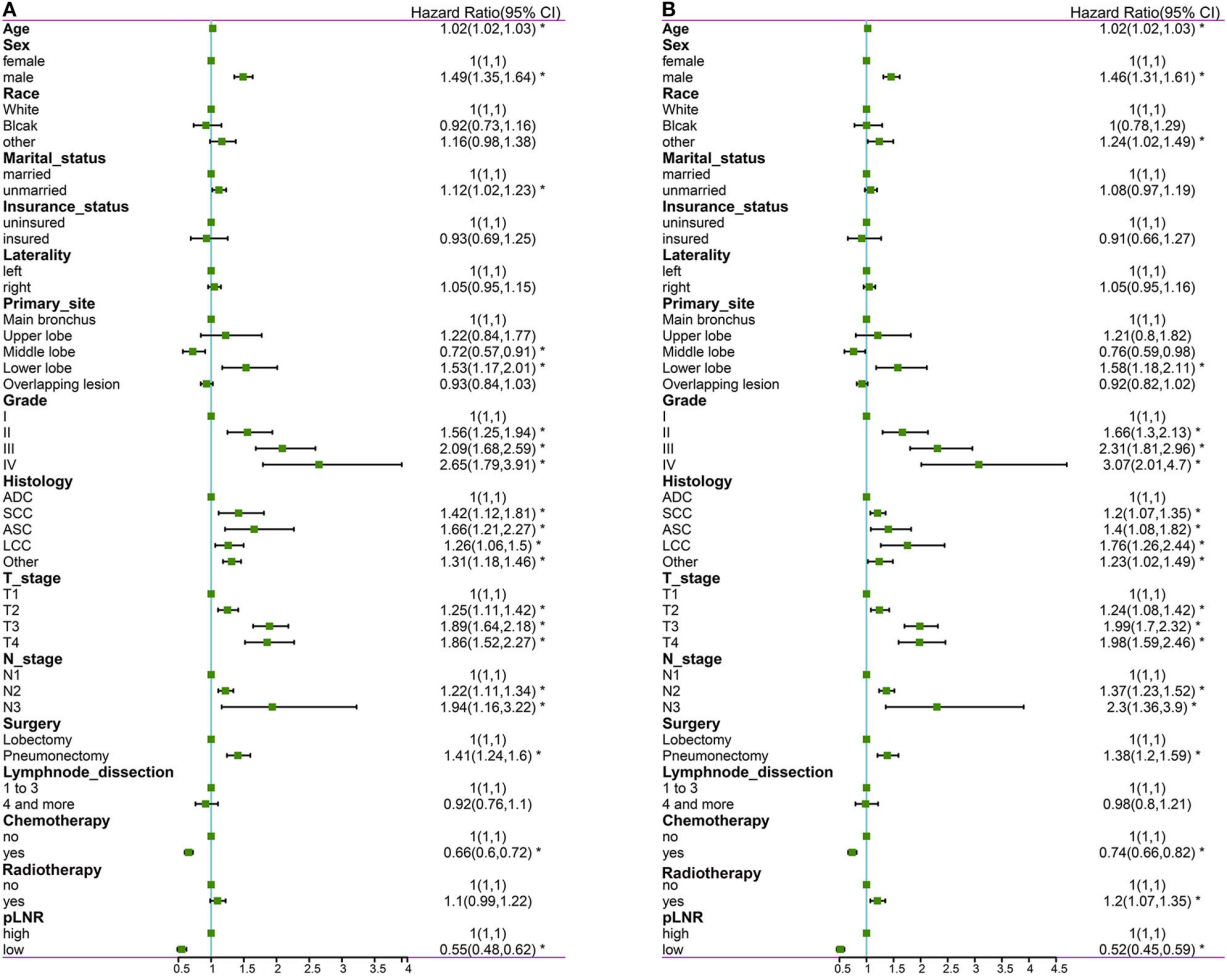
The forest map of Cox regression analysis. Univariate Cox regression analyses estimating the risk factors for OS **(A)** and CSS **(B)**. OS, overall survival; CSS, cancer-specific survival. *Means *P* < 0.05.

**Figure 3:**
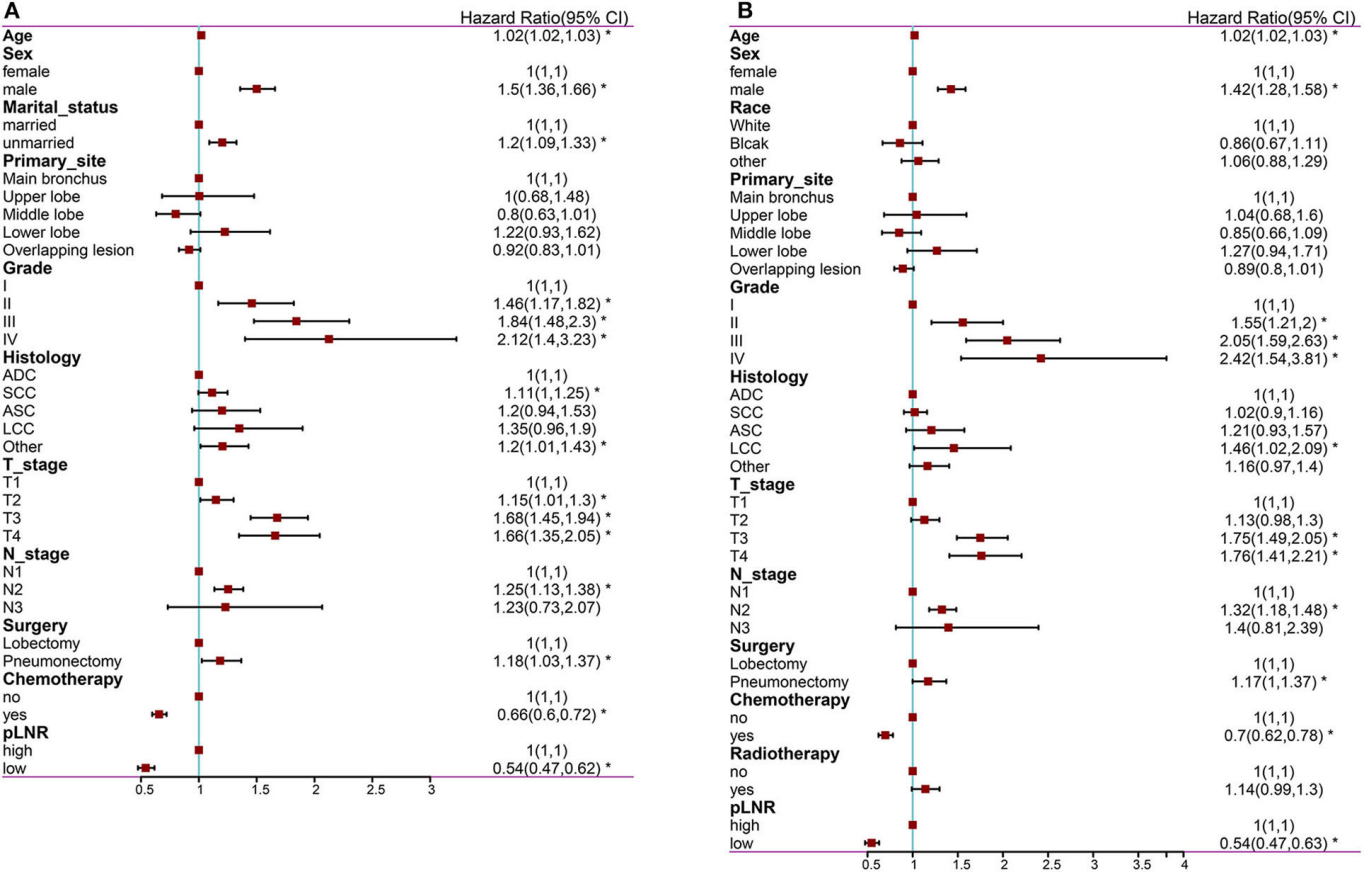
The forest map of Cox regression analysis. Multivariate Cox regression analyses estimating the risk factors for OS **(A)** and CSS **(B)**. OS, overall survival; CSS, cancer-specific survival. *Means *P* < 0.05.

The following factors were included in the univariate Cox regression analysis: age, race (White vs. Black vs. other), sex (male vs. female), marital status (married vs. unmarried), insurance status (insured vs. uninsured), histological type (ADC vs. SCC vs. ASC vs. LCC vs. other), grade (well differentiated, grade I vs. moderately differentiated, grade II vs. poorly differentiated, grade III vs. undifferentiated or anaplastic, grade IV), primary site (main bronchus vs. upper lobe vs. middle lobe vs. lower lobe), laterality (left vs. right), T stage (T1 vs. T2 vs. T3 vs. T4), N stage (N1 vs. N2 vs. N3), surgery at the primary site (lobectomy vs. pneumonectomy), scope of regional lymph node surgery (1-3 regional lymph nodes removed vs. ≥4 regional lymph nodes removed), radiation therapy (yes vs. no), chemotherapy (yes vs. no) and pLNR (high vs. low). The prognostic factors with significant differences were included in the multivariate Cox regression analyses for OS and CSS. The multivariate Cox regression analyses revealed that primary site was not significant for OS and that race, primary site, surgery and radiation therapy were not significant for CSS. The other prognostic factors were included in the construction of the nomogram. The results of the Cox regression analysis of OS are shown in Figures 2A, 3A, and those for CSS are shown in Figures 2B, 3B.

**Figure 4:**
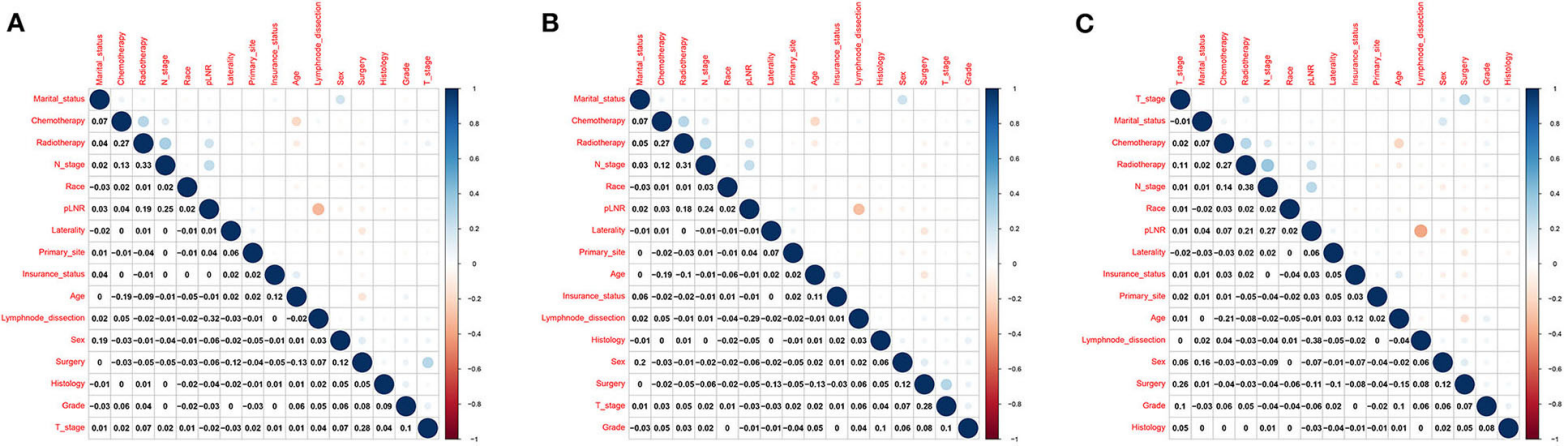
Correlations between variables in the overall dataset. **(A)** The training set **(B)** and the validation set **(C)**.

There was no significant correlation among N stage, the pLNR and the other independent variables for the overall dataset, the training set or the validation set (Figure 4). Furthermore, the tolerance was >1, and VIF was significantly <10 for the overall dataset, the training set and the validation set (Supplementary Table 1), indicating no collinearity among the independent variables.

### Construction and Validation of the Nomogram

**Figure 5:**
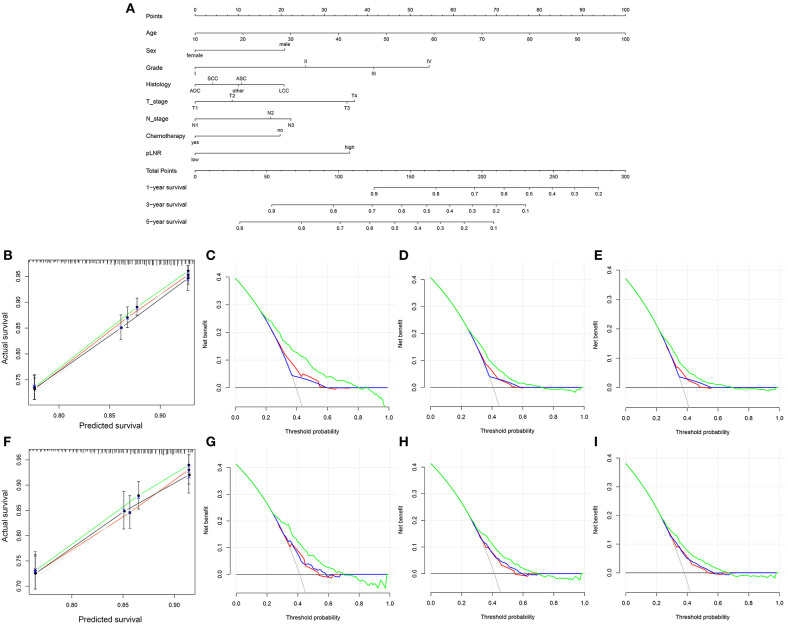
**(A)** Nomogram used to predict the 1-, 3- and 5-year OS rates of patients with **T_1−4_N_1−3_M_0_** NSCLC. **(B)** Calibration curve of the nomogram for predicting the 1-, 3- and 5-year OS rates of patients with **T_1−4_N_1−3_M_0_** NSCLC from the training set. Decision curve analysis of the AJCC 7th stage, nomogram and positive lymph node ratio (pLNR) for the 1- **(C)**, 3- **(D)** and 5-year **(E)** OS rates of patients with **T_1−4_N_1−3_M_0_** NSCLC from the training set. **(F)** Calibration curve of the nomogram for predicting the 1-, 3- and 5-year OS rates of patients with **T_1−4_N_1−3_M_0_** NSCLC from the validation set. Decision curve analysis of the AJCC 7th stage, nomogram and pLNR for the 1- **(G)**, 3- **(H)** and 5-year **(I)** OS rates of patients with **T_1−4_N_1−3_M_0_** NSCLC from the validation set. OS, overall survival; pLNR, positive lymph node ratio; NSCLC, non-small cell lung cancer. For calibration curves, green, red, and black line represent 1, 3, and 5 years, respectively. For decision curve analysis, green represents the nomogram, red represents pLNR, and blue represents AJCC 7th stage.

**Figure 6:**
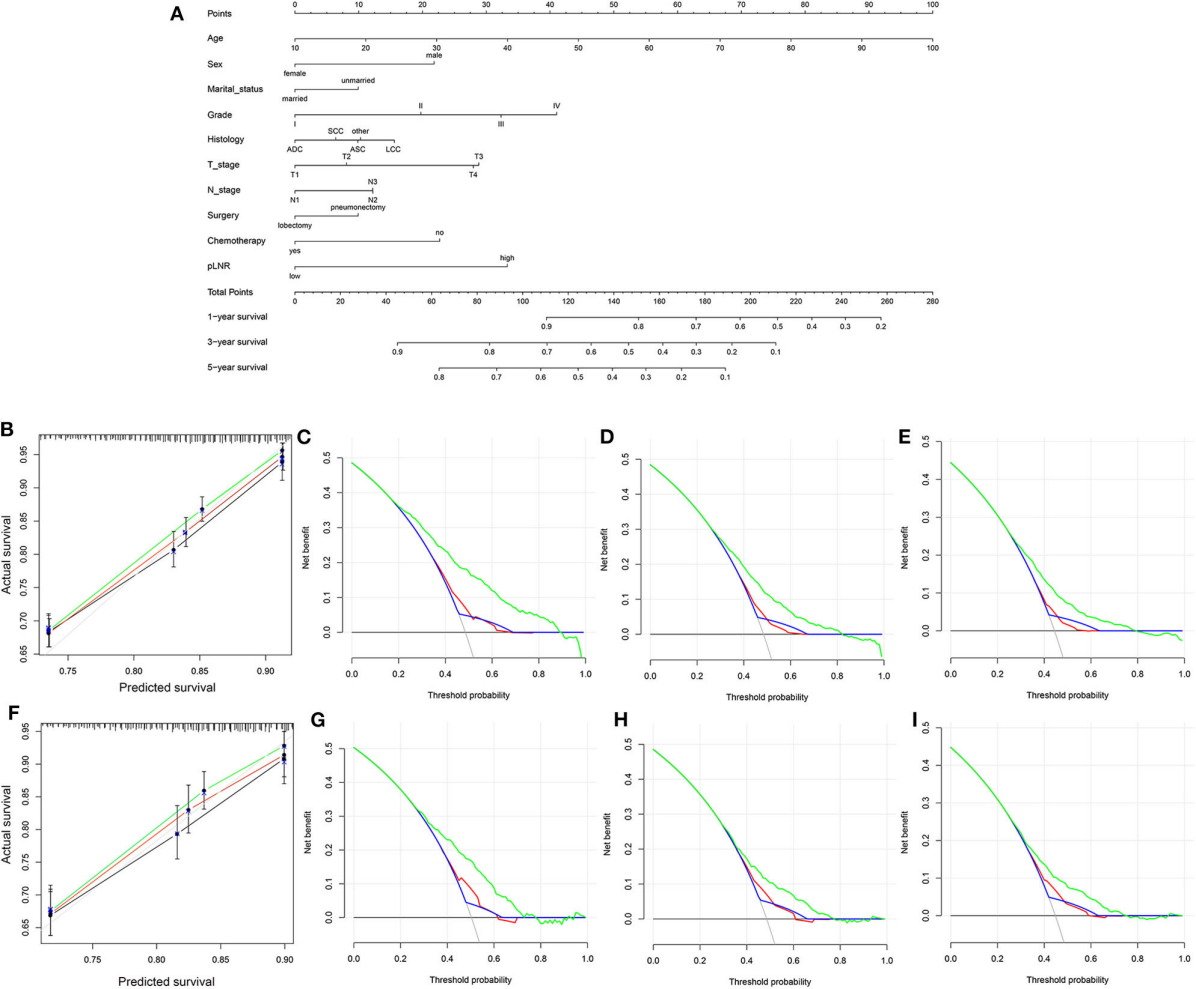
**(A)** Nomogram used to predict the 1-, 3-, and 5-year CSS rates of patients with **T_1−4_N_1−3_M_0_** NSCLC. **(B)** Calibration curve of the nomogram for predicting the 1-, 3-, and 5-year CSS rates of patients with **T_1−4_N_1−3_M_0_** NSCLC from the training set. Decision curve analysis of the AJCC 7th stage, nomogram and positive lymph node ratio (pLNR) for the 1- **(C)**, 3- **(D)** and 5-year **(E)** CSS rates of patients with **T_1−4_N_1−3_M_0_** NSCLC from the training set. **(F)** Calibration curve of the nomogram for predicting the 1-, 3-, and 5-year CSS rates of patients with **T_1−4_N_1−3_M_0_** NSCLC from the validation set. Decision curve analysis of the AJCC 7th stage, nomogram and pLNR for the 1- **(G)**, 3- **(H)**, and 5-year **(I)** CSS rates of patients with **T_1−4_N_1−3_M_0_** NSCLC from the validation set. CSS, cancer-specific survival; pLNR, positive lymph node ratio; NSCLC, non-small cell lung cancer. For calibration curves, green, red, and black line represent 1, 3, and 5 years, respectively. For decision curve analysis, green represents the nomogram, red represents pLNR, and blue represents AJCC 7th stage.

The nomogram for OS comprised 10 prognostic factors: age, sex, marital status, histological type, grade, T stage, N stage, surgery type, chemotherapy and pLNR (Figure 5A). The nomogram for CSS included 8 prognostic factors: age, sex, histological type, grade, T stage, N stage, chemotherapy and pLNR (Figure 6A). We concluded that the pLNR plays an important role in patient prognosis. The total score was calculated by adding the scores of each factor according to the clinical characteristics, and the 1-, 3-, and 5-year survival rates were estimated by drawing a straight line from the total score on the nomogram. For the training set, the C-index, calculated by the bootstrap self-sampling method, was 0.681 for OS and 0.673 for CSS. For the validation set, the C-index was 0.674 for OS and 0.678 for CSS. The predicted calibration curves were close to the standard curves for 1-, 3-, and 5-year survival for both OS (Figure 5B) and CSS (Figure 6B) in the training set and for both OS (Figure 5F) and CSS (Figure 6F) in the validation set. For both the training and validation sets, the DCA curves for OS (Figures 5C–E,G–I) and CSS (Figures 6C–E,G–I) indicated that the pLNR had a good predictive ability regarding patient prognosis, and the predictive power of our nomogram was better than that of the AJCC staging system (7th edition).

### Risk Stratification

**Figure 7:**
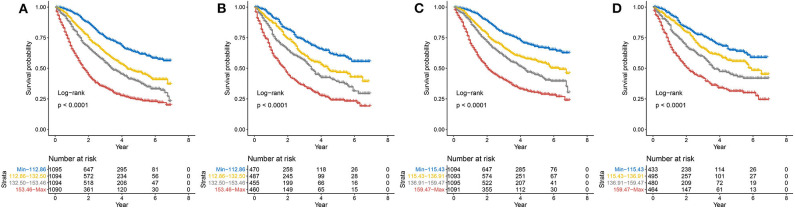
Kaplan–Meier curves of risk group stratification within the training set for **(A)** OS and **(B)** CSS and within the validation set for **(C)** OS and **(D)** CSS. OS, overall survival; CSS, cancer-specific survival.

The total score was calculated for each patient in the training and validation sets, and the scores were divided into quartiles for OS (Min-112.86, 112.86–132.50, 132.50–153.46, 153.46-Max) and CSS (Min-115.43, 115.43–136.91, 136.91–159.47, 159.47-Max) to represent different outcomes. Statistically significant differences in OS (Figures 7A,C) and CSS (Figures 7B,D) were observed after stratifying patients according to quartile (all *P* < 0.001).

## Discussion

In this study, we screened data from the SEER database according to inclusion and exclusion criteria and conducted univariate and multivariate Cox regression analyses to identify prognostic risk factors. We conclude that the pLNR is a significant factor influencing survival of **T_1−4_N_1−3_M_0_** NSCLC patients and can be used to predict patient prognosis. Visualization of the analysis results of the multiple risk factors with the nomogram proved the significant influence of the pLNR on prognosis. The C-index values, calibration curves and DCA curves also proved the good clinical predictive ability of the nomogram. Furthermore, risk stratification also proved the clinical applicability of the nomogram for patients of different stages.

To effectively and accurately treat NSCLC patients, all the prognostic factors that may affect survival should be considered. At present, the AJCC staging of lung cancer (eighth edition) is based mainly on the anatomical locations of lymph nodes and does not consider the number or proportion of positive lymph nodes. However, as early as the release of the 7th edition of the AJCC guidelines, Bria et al. (17) reviewed 415 NSCLC patients in Italy and indicated that the pLNR was an independent prognostic factor. Our current nomogram also shows that the pLNR has a significant influence on prognosis, so it is necessary to study the prognostic value of the pLNR.

Additionally, research using the SEER database has confirmed the ability of the pLNR to predict the survival of NSCLC patients. For example, Urban et al. (18) found that a high pLNR was associated with poor survival for patients with resected, node-positive (N1-N2) NSCLC. Ding et al. (19) analyzed data from 109,026 NSCLC patients and found that the pLNR had better predictive ability than N stage regarding patient survival following radiotherapy. Deng et al. (20) found that the pLNR had good predictive value for non-advanced NSCLC patients, both in terms of OS and cancer-specific survival (CCS). In a recent study, Han et al. (21) confirmed that the pLNR combined with TNM stage could predict the prognosis of patients with IIIa-N2 NSCLC.

The results of a number of clinical retrospective studies from all over the world, including studies on different ethnic groups, are consistent with our results based on the SEER database. A meta-analysis of five retrospective studies representing 6,130 non-advanced NSCLC patients from the United States and China indicated that the pLNR could be used to predict the OS and disease-free survival (DFS) of patients and detected no publication bias (22). According to clinical research, the pLNR can be used to assess not only the prognosis of early-stage NSCLC patients but also the risk of brain metastasis in late-stage NSCLC patients (23). Moreover, the pLNR can be used to predict the effects of postoperative radiotherapy and chemotherapy in NSCLC patients (24).

The appropriate partitioning of pLNR cut-off values is debated, and different studies have adopted different methods to determine the truncation value of the pLNR. Matsuguma et al. (25) divided the pLNR into three levels (0, 0.01 ~ 0.12 and >0.12) and used the median of the different groups as the truncated value. In another study on pN1 NSCLC patients, Bria et al. (17) used a classification and regression tree (C&RT) analysis and determined that 9% was the optimal cut-off value for the pLNR based on the maximum log-rank statistical value. The Youden index (26) of the ROC curve can be used to achieve the general dichotomous outcome index, but for survival data, it is difficult to determine the cut-off value. The innovation of our study was that we used X-tile software to determine the minimum *P*-value of the two groups of data (high and low pLNR) to identify the optimal truncation value.

The pLNR was not the only prognostic factor included in our nomogram. Treatment was another independent prognostic factor, and chemotherapy was identified as beneficial to patient survival. However, expanded resection, such as pneumonectomy, did not improve prognosis over lobectomy in our study. Similar findings were reported by Anderson et al. (27), who analyzed the 5-year survival rate of 641 patients with T4 NSCLC. Although they found no significant difference in survival between patients who underwent two different types of surgery, patients who underwent lobectomy did not die after 5 years. However, some scholars believe that pneumonectomy does not lead to a worse prognosis than lobectomy and that pneumonectomy should remain an option for certain patients (28). Currently, the specific scope of lymph node dissection remains controversial. Expanded regional lymph node dissection does not benefit stage I NSCLC patients, whereas for patients after stage I, such measures can significantly improve prognosis (29). Whether radiotherapy has a beneficial effect on the prognosis of patients is debated. In particular, the reported effects of radiotherapy on prognosis for different stages of lung cancer are inconsistent (30, 31).

Our nomogram revealed additional prognostic demographic factors, such as age, sex, and marital status. Due to the declining health of elderly patients, cancer resistance in such patients is poorer than that in young patients; thus, prognosis in these patients is poor (32, 33). Although the death rate of women with lung cancer is increasing, it remains lower than that of men with lung cancer (1). Further research is needed to determine the mechanism involved. Regarding race, a study from Florida showed that Asians have better prognosis than Blacks and Whites, which supports our hypothesis (34). However, the majority of the remainder of the population in the SEER database is Asian. Furthermore, a 10-year follow-up survey conducted by the Veteran Affairs Central Cancer Registry in the United States showed that ethnic differences did not affect survival or CSS (35). Interestingly, our study found that marital status had some impact on the prognosis of nonadvanced NSCLC patients. Galvin et al. (36) found that marriage significantly reduced the mortality rate of women in their study and that the prognosis of cancer patients was influenced by social and psychological factors.

NSCLC is a heterogeneous disease, and personalized treatment is very important. Therefore, it is necessary to identify prognostic factors to improve the survival rate of patients. The nomogram established in this study has not only high predictive power but also significance for clinical treatment. For example, according to the demographic and clinicopathologic characteristics of patients, scores can be obtained, and survival rates can be estimated. When the estimated survival rate of a patients is low, the choice of follow-up treatment, especially surgical treatment, should be made with care.

To our knowledge, the present study is the first retrospective study of a large number of NSCLC patients with extensive staging and the first to use the pLNR as a prognostic marker to construct a nomogram. However, our research has some shortcomings. First, our study is a retrospective study, and some prognostic factors that were not included in our study due to the limitations of the SEER database, such as smoking history, family history, receipt of targeted therapy or immunotherapy and type of lymph node involvement, may have affected our results. Additionally, the staging system that we used was the 7th edition of the AJCC staging system, and since the SEER database provides tumor size but not the specific site of the tumor or whether it had invaded the prostate or surrounding organs, we were unable to classify tumor information according to the 8th edition of the AJCC staging system. Finally, the SEER database contains data on patients in the United States, which, although abundant, are probably not very representative of lung cancer patients worldwide.

## Conclusions

The pLNR is an independent risk factor for non-advanced NSCLC. A nomogram combining demographic, pathological and treatment data was established to predict OS and CSS for patients with non-advanced NSCLC and validated using data from the SEER database.

## Data Availability Statement

Publicly available datasets were analyzed in this study. This data can be found here: https://seer.cancer.gov/data/.

## Author Contributions

YL and GY conceived and designed the study, acquired and analyzed the data and wrote the manuscript. XF contributed to data analysis and manuscript preparation. All authors read and approved the manuscript and agree to be accountable for all aspects of the research in ensuring that the accuracy or integrity of any part of the work is appropriately investigated and resolved.

## Conflict of Interest

The authors declare that the research was conducted in the absence of any commercial or financial relationships that could be construed as a potential conflict of interest.
