# Supplementary material for: The Positive Lymph Node Ratio Predicts Survival in T1−4N1−3M0 Non-Small Cell Lung Cancer: A Nomogram Using the SEER Database
Source: Front Oncol. 2020 Aug 5;10:1356. doi: 10.3389/fonc.2020.01356 (PMC7438846; doi:10.3389/fonc.2020.01356)
Supplement: Supplementary file 1 [file Table_1.DOC]

Supplymentary table 1 The tolerance and VIF for the overall dataset, the training set and the validation set, pLNR is regarded as the dependent variable, and the remaining influencing factors are independent variables. VIF, variance inflation factor; pLNR, positive lymph node ratio.

|  | overall | | Training set | | Validation set | |
| --- | --- | --- | --- | --- | --- | --- |
| Characterictics | tolerance | VIF | tolerance | VIF | tolerance | VIF |
| Laterality | 0.982 | 1.018 | 0.979 | 1.021 | 0.98 | 1.02 |
| Race | 0.993 | 1.007 | 0.992 | 1.008 | 0.988 | 1.012 |
| Sex | 0.938 | 1.067 | 0.931 | 1.074 | 0.942 | 1.062 |
| Primary_site | 0.988 | 1.012 | 0.987 | 1.013 | 0.987 | 1.013 |
| Grade | 0.965 | 1.036 | 0.966 | 1.036 | 0.95 | 1.052 |
| Histology | 0.986 | 1.014 | 0.983 | 1.017 | 0.989 | 1.011 |
| T_stage | 0.905 | 1.105 | 0.902 | 1.109 | 0.903 | 1.108 |
| N_stage | 0.887 | 1.128 | 0.899 | 1.113 | 0.844 | 1.185 |
| Surgery | 0.866 | 1.155 | 0.862 | 1.16 | 0.865 | 1.156 |
| Radiation | 0.828 | 1.208 | 0.84 | 1.19 | 0.788 | 1.27 |
| Chemotherapy | 0.885 | 1.13 | 0.886 | 1.128 | 0.868 | 1.152 |
| Insurance_status | 0.982 | 1.018 | 0.982 | 1.019 | 0.971 | 1.03 |
| Marital_status | 0.951 | 1.052 | 0.943 | 1.06 | 0.964 | 1.038 |
| Age | 0.915 | 1.093 | 0.921 | 1.086 | 0.897 | 1.115 |
| Lymphnode_dissection | 0.988 | 1.012 | 0.987 | 1.013 | 0.981 | 1.019 |
